# Impact of HIV-1 Vpr manipulation of the DNA repair enzyme UNG2 on B lymphocyte class switch recombination

**DOI:** 10.1186/s12967-020-02478-7

**Published:** 2020-08-10

**Authors:** Patrick Eldin, Sophie Péron, Anastasia Galashevskaya, Nicolas Denis-Lagache, Michel Cogné, Geir Slupphaug, Laurence Briant

**Affiliations:** 1 Institut de Recherche en Infectiologie de Montpellier (IRIM), CNRS, UMR 9004, Université de Montpellier, 1919 Route de Mende, 34293 Montpellier Cedex 5, France; 2 Contrôle de la Réponse Immune B et des Lymphoproliférations (CBRIL), UMR CNRS 7276 INSERM 1262, Centre de Biologie et de Recherche en Santé (CBRS), Faculté de Limoges, 2 rue du Dr. Marcland, 87000 Limoges, France; 3 Proteomics and Modomics Experimental Core (PROMEC), Department of Cancer Research and Molecular Medicine, Laboratory Centre, Norwegian University of Science and Technology (NTNU), 5th Floor. Erling Skjalgssons gt. 1, 7491 Trondheim, Norway

**Keywords:** Uracil DNA glycosylase 2, Human immunodeficiency virus, Vpr, Uracilation, Class switch recombination

## Abstract

**Background:**

HIV-1 Vpr encodes a 14 kDa protein that has been implicated in viral pathogenesis through modulation of several host cell functions. In addition to pro-apoptotic and cytostatic properties, Vpr can redirect cellular E3 ubiquitin ligases (such as DCAF1-Cul4A E3 ligase complex) to target many host proteins and interfere with their functions. Among them, Vpr binds the uracil DNA glycosylase UNG2, which controls genome uracilation, and induces its specific degradation leading to loss of uracil removal activity in infected cells. Considering the essential role of UNG2 in antibody diversification in B-cells, we evaluated the impact of Vpr on UNG2 fate in B lymphocytes and examined the functional consequences of UNG2 modulations on class switch recombination (CSR).

**Methods:**

The impact of Vpr-induced UNG2 deregulation on CSR proficiency was evaluated by using virus-like particles able to deliver Vpr protein to target cells including the murine model CSR B cell line CH12F3 and mouse primary B-cells. Co-culture experiments were used to re-examine the ability of Vpr to be released by HIV-1 infected cells and to effectively accumulate in bystander B-cells. Vpr-mediated UNG2 modulations were monitored by following UNG2 protein abundance and uracil removal enzymatic activity.

**Results:**

In this study we report the ability of Vpr to reduce immunoglobulin class switch recombination (CSR) in immortalized and primary mouse B-cells through the degradation of UNG2. We also emphasize that Vpr is released by producing cells and penetrates bystander B lymphocytes.

**Conclusions:**

This work therefore opens up new perspectives to study alterations of the B-cell response by using Vpr as a specific CSR blocking tool. Moreover, our results raise the question of whether extracellular HIV-1 Vpr detected in some patients may manipulate the antibody diversification process that engineers an adapted response against pathogenic intruders and thereby contribute to the intrinsic B-cell humoral defect reported in infected patients.

## Background

The Vpr HIV-1 accessory protein that possesses plethoric functions during the HIV life cycle has a pivotal role in viral pathogenesis and progression towards AIDS (for review see [1]). Briefly, Vpr enhances viral replication by 2–4 fold in proliferating T-cells [2] and its expression is essential for efficient viral replication in non-dividing cells such as macrophages [3]. These effects were ascribed to the capacity of Vpr to facilitate the cytoplasmic-nuclear shuttling of reverse transcription complexes and viral genome nuclear import during early infection events [4]. It also reflects the Vpr-mediated stimulation of HIV-1 gene transcription either directly by interacting with several transcription factors [5] or indirectly, for example, by enhancing phosphorylation and polyubiquitination of TAK1 and subsequent NF-κB/AP-1-dependent HIV-1 LTR stimulation [6]. The biochemical mechanisms mediating Vpr function in HIV-1 infection are mainly related to Vpr ability to usurp the Cul4-DDB1[VprBP] E3 ubiquitin ligase complex comprising the Cullin 4 (Cul4) scaffold, DDB1 linker protein, DDA1 core subunit [7], and DCAF1 substrate receptor [8, 9], to which Vpr binds [10]. Vpr also induces cell cycle arrest of infected cells at the G2/M phase [11]. This last extensively described function [12], has been initially associated with the capacity of Vpr to activate the ataxia telangiectasia-mutated (ATM) [13] and Rad3-related proteins (ATR) and to stimulate phosphorylation of ATR substrates Chk1 and H2AX histone variants [14]. But recently, this cytostatic property has been correlated with the activation of the structure-specific endonuclease regulator SLX4 complex by Vpr [15]. Finally, Vpr also induces apoptosis through diverse mechanisms, either related to mitochondrial dysfunction, or by interacting with Bax or the voltage-dependent anion channel (VDAC), all leading to cytochrome *c* release and caspase 3 activation [16].

Exploration of Vpr-recruited substrates identified proteins involved in epigenetic control of gene expression, such as ten eleven translocation methylcytosine dioxygenase 2 (Tet2), and key factors in DNA damage response and repair [17], mainly MUS81 [15], helicase like transcription factor (HLTF) [18], and uracil-DNA glycosylase 2 (UNG2) [19]. More specifically, UNG2, the nuclear isoform of UNG, excises uracil from DNA that results from misincorporation of dUMP by DNA polymerase or from cytosine deamination, thus initiating base excision repair [20]. In keeping with our initial identification of Vpr activation of HIV-1 LTR transcription via UNG2 proteasome-dependent degradation that counteracts UNG2 anti-transcriptional activity [21], we have recently associated this Vpr-mediated mechanism with a substantial increase in genomic uracilation in HIV-1 infected T-cells [22]. Besides its major involvement in the base excision DNA repair pathway (BER) required for uracil removal from DNA and preservation of genome integrity in all cell types, UNG2 also plays a specific crucial role in antibody diversification in B-cells [23, 24], by excising uracil derived from cytosine deamination by activation-induced deaminase (AID) [25]. This is an essential step in somatic hypermutation (SHM) and class switch recombination (CSR) that generates antibodies with increased antigen affinity and expanded effector functions, respectively [26].

Here, we present a proof of concept study designed to evaluate the ability of Vpr to alter B cell functions through UNG2 manipulation. Using an approach aimed to drive Vpr entry in target B-cells, we evaluated its impact on B-cell uracil excision and CSR progression. We found that Vpr in human immortalized B-cells was competent to induce UNG2 degradation in a proteasome-dependent manner, leading to a decrease in UNG activity and an increase in genome uracilation, without significantly affecting cell cycle and viability. To monitor the impact of Vpr-induced UNG2 deregulation on CSR proficiency, these experiments were transposed to the CH12F3 murine B-cell line, widely used as a model system for CSR studies in which UNG2 expression and enzyme activity were reproducibly decreased by Vpr delivery. Using this model, we report the capacity of Vpr to decrease CSR capacities of stimulated mouse CH12F3 cells, a result that was further validated in primary B-cells. B-cells are non-permissive to HIV-1 infection. However, Vpr is endowed with cell penetrating properties [27, 28] and in some studies was reported in an extracellular phase in the blood of HIV-1-infected patients [29]. Here we demonstrate that the ability of Vpr expressed by producing cells to accumulate in bystander human and mouse B-cells, leads to proteasome-mediated UNG2 depletion. Our results therefore highlight the potential of cell-penetrating Vpr to manipulate UNG2 in B-cells, thereby dampening CSR and potentially contributing to HIV-1-induced humoral response deficiency.

Results are discussed in terms of the perspectives uncovered by these preliminary data suggesting a potential direct impact of Vpr-mediated loss of UNG2 function in antibody diversification, and its possible contribution to B-cell intrinsic defects reported in HIV-1 infected patients in which Vpr can be released by infected cells and penetrate bystander B lymphocytes.

## Methods

### Reagents and antibodies

The following antibodies were used: rabbit polyclonal anti-human UNG2 clone PU59 and rabbit anti-mouse UNG2 clone PUMA have been previously described [30, 31]. The latter was used to immunoprecipitate mouse UNG2 which cannot be directly detected by PU59 antibody. Anti-hUNG2 rabbit polyclonal NBP1-49985 was from Novus Biologicals; anti-Vpr mixture was made with equal amounts of goat polyclonal antibody (sc-17493) from Santa Cruz Biotechnology, rabbit polyclonal antibody #709 and #11836 (both from NIH AIDS reagent program); anti-α tubulin mouse monoclonal antibody (T5168) was from Sigma; goat polyclonal anti-p24 (4999-9007) was from AbD Serotec; Secondary HRP-conjugated antibodies were from Jackson Laboratory. UGI inhibitor was purchased from New England Biolabs. MG132 was from Sigma Aldrich.

### Plasmids

**Fig. 1:**
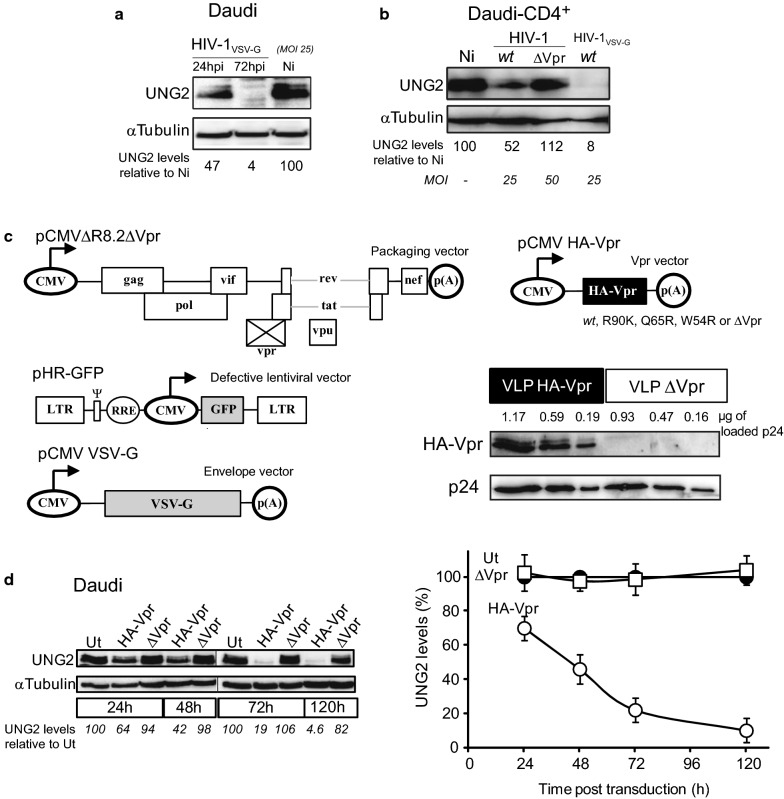
HA-Vpr-delivering VLPs decrease UNG2 expression in human B lymphocytes. **a** Daudi cells were infected with VSV-G pseudotyped *wt* HIV-1 at MOI of 25. 24 and 72 h after infection UNG2 levels were examined by immunoblot with anti-UNG2 antibody or anti-α-tubulin as loading control. **b** Daudi-CD4 + cells were infected with either HIV-1 *wt*, HIV-1ΔVpr or VSV-G pseudotyped *wt* HIV-1 at the indicated MOI. 72 h after infection UNG2 levels were examined by immunoblot with anti-UNG2 antibody or anti-α-tubulin as loading control (Ni: non-infected). **c** HA-Vpr-delivering VLPs were generated by cotransfecting HEK 293T cells with pCMV HA-Vpr along with a packaging vector pCMVΔR8.2ΔVpr, an Envelope vector pCMV VSV-G and the defective lentiviral vector pHR-GFP. VLPs produced in the presence (VLP HA-Vpr) or absence of pCMV HA-Vpr (VLP ΔVpr) were purified on sucrose gradients, and titrated by p24 ELISA kit. VLP titers were also determined by transducing 293T cells with serial dilutions of virus suspensions. Virus extracts corresponding to the indicated p24 amount were resolved on PAGE-SDS gels, and immunoblotted with specific antibodies for their content in p24 and HA-Vpr. **d** Daudi B-cells were transduced with VLP HA-Vpr or ΔVpr at M.O.I of 10. Cells were lysed at different time points and examined by immunoblot for UNG2 content with anti-UNG2 antibody or anti-α-tubulin as loading control. Lanes were regrouped from two different gels, see Supplementary dataset for detail. UNG2 levels at the various time points were quantified with ImageJ and normalized with their corresponding α-tubulin levels and plotted as a UNG2% of untransduced (Ut) cells. Lanes were cropped from blot membranes serially probed with anti-UNG2 then with anti-αtubulin antibodies (see Additional file 2: Supplementary dataset 2)

The pHR-Vpr (*wt* and mutants), pHR-GFP, pCMVΔR8.2ΔVpr, pCMV-VSV-G constructs (see Figs. 1c and  6a) were kindly provided by Vicente Planelles (University of Utah School of Medicine, Salt Lake City, USA). pCMV HA-Vpr construct was generated by cloning a PCR-derived HA-Vpr amplicon derived from pNL4.3 Vpr as template in pcDNA3.1. Plasmids encoding for viral molecular clones pNL4.3 and pNL4.3ΔVpr (from W.C. Greene, Gladstone Institutes San Francisco, USA) were used to produce HIV-1 and HIV-1 ΔVpr viruses, respectively.

### Mice

Mice were maintained in our animal facilities (BISCEm (Research Facility for Integrative Biology, Health, Chemistry and Environment), Limoges) approved by *French Ministry of Agriculture* (No A8708505), under specific-pathogen-free conditions. Mice were housed and procedures were conducted in agreement with *European Directive 2010/63/EU on animals used for scientific purposes* applied in France as stipulated in the “*Décret no2013*-*118 du 1*^*er*^ *Février 2013 relatif à la protection des animaux utilisés à des fins scientifiques*”. All methods in the current study were carried out in accordance with relevant guidelines and regulations and all experimental protocols were approved by French institutions. The experimental protocols were specifically approved by *Comité Régional Ethique Animale du Limousin no33* and authorized by *Ministère de l’Éducation Nationale, de l’Enseignement Supérieur et de la Recherche/Plateforme* Autorisation-Projet website Recherche.gouv.fr (APAFIS #16216-2018072015045506 v3).

### Cell culture

CH12F3-2 (from T. Honjo, Kyoto, Japan), and Daudi B-cells were maintained in RPMI 1640 complete medium. HEK 293T cells were maintained in DMEM complete medium. Complete media were supplemented with penicillin–streptomycin (Lonza) and 10% heat-inactivated fetal bovine serum (FBS) (Cambrex). Primary B-cells were prepared from 5- to 6-weeks old C57BL/6 mice (Charles River, USA) or from AID^−/−^ mice (formally *Aicda*^−/−^) [32, 33] provided by François Huetz (Pasteur Institute, Paris), as follows. Erythrocyte-depleted mouse spleen cells were cultured in RPMI medium supplemented with 10% FBS, sodium pyruvate, nonessential amino acids, 50 µM β-mercaptoethanol, 100 U/mL penicillin, and 100 μg/mL streptomycin (Invitrogen). Mature resting B-cells were isolated by depletion of CD43-expressing B-cells (Myltenyi Biotec).

### Co-culture experiments

In a 6-well plate format, HEK 293T cells (3 × 10^5^ cells per well) were transduced with lentiviral vectors driving (Vpr+) or not (Vpr-) the expression of Vpr (produced as outlined in Fig. 1c) at a M.O.I. of 10. Efficient integration and expression was visualized 72 h later by the expression of the GFP reporter by both VLPs (Fig. 6a). Cells were then washed and co-cultured with Daudi B-cells (2 × 10^6^ cells per well) suspended on top of transduced HEK 293T layers in cell culture Millicell hanging inserts with hydrophilic 0.4 µm PTFE membranes (MerckMillipore) (see Fig. 6b). Daudi cells were immobilized on poly(l-lysine)-coated cover slips 72 h later, formalin-fixed and labeled with DAPI and anti-Vpr antibodies to visualize nuclei and Vpr, respectively. In parallel, whole cell extracts were prepared from Daudi and HEK 293T cells and UNG activity was measured (see below in *Uracil DNA glycosylase assays* section) and Vpr immunoprecipitation. Briefly, cells were lysed in 50 mM Tris–HCl (pH 8.0), 100 mM NaCl, 1 mM MgCl_2_, 1% Triton X-100 and protease inhibitors (Complete, Roche). Fifty μg of total protein was kept as input and the remaining cell lysate (150 µg) was incubated with antibodies for 2 h at 4 °C and with Protein A/G Magnetic Beads (Thermo Fisher) overnight at 4 °C. The beads/immune complexes were washed 6 times with 1 mL lysis buffer and released from the beads by boiling in 1 × Laemmli buffer. UNG2 and Vpr contents were analyzed in input and Vpr IP samples, respectively. The same 6 well format was used to perform the co-culture experiment with MAGIC5B producer cells infected with either HIV-1 *wt* or HIV-1ΔVpr and Daudi as bystander cells maintained in cell inserts (Fig. 6e).

### Immunofluorescence

Daudi cells were adsorbed on polyLysine-treated slides and fixed with Formalin (Sigma) containing 1% Triton-X-100. Cells were labeled with anti-Vpr antibody followed by labeling with a mixture of Alexa488-conjugated donkey anti-goat and anti-rabbit IgG (H + L) antibodies (Life Technologies). Nuclei were stained with DAPI staining solution from Sigma Aldrich. Cells were mounted on glass slides covered with anti-fade medium (Hardset Vectashield). Two-color images were obtained with a light microscope, Leica DC250 (Leica) with a Plan Apo 63 ×/1.32–0.6 oil-immersion objective lens. Digital images were processed with the ImageJ software (NIH Image). Percentages of Vpr^+^ Daudi B-cells were determined by using the Cell Counting plugin of ImageJ. A minimum of 750 cells were counted per 6-well plate.

### Cell cycle and apoptosis

Cells were collected, washed twice and resuspended in ice cold PBS. Then the cells were fixed with 70% EtOH for 1 h on ice. Cells were pelleted for 5 min at 1500 rpm, washed twice with PBS and resuspended in a solution containing 50 µg/mL propidium iodide, 0.1 mg/mL RNase A, 0.1% Triton X-100 in PBS. After 30 min incubation at 4 °C in the dark, DNA content was determined by flow cytometry using an EPICS PROFILE XL4C cytofluorometer (Coulter). Appropriate gating was applied to discriminate singlets and aggregated cells. Curve fitting decomposition was done with FCS Express (De Novo Software). For apoptosis evaluation, cells were incubated in PBS containing CaCl2 (0.33 g/L) and labeled with Annexin V-FITC (Immunotools) for 15 min at room temperature and analyzed by flow cytometry. Cell viability was performed by using the MTT viability assay Cell Titer™ (Promega).

### Viral stock production

**Fig. 2:**
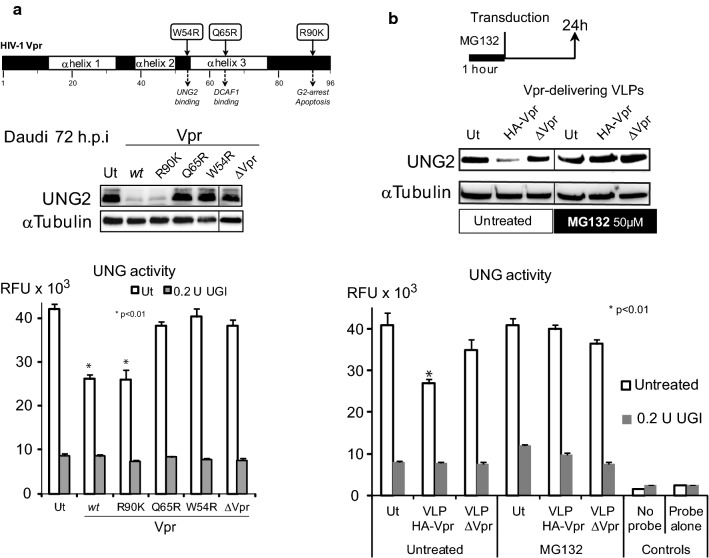
VLP-delivered HA-Vpr induces a proteasome-dependent decrease in human B lymphocyte uracil removal capacity. **a** Daudi B-cells were transduced with VLP HA-Vpr *wt* or mutant (R90K, Q65R or W54R) or ΔVpr at M.O.I of 10. 72 h later cells were lysed and examined by immunoblot for UNG2 content (with anti-UNG2 antibody or anti-α tubulin as control). Lanes were cropped from different parts of a unique gel (see Additional file 2: Supplementary dataset 2). Corresponding UNG activity was measured in the presence or absence of the UNG inhibitor UGI. **b** Daudi cells were treated for 1 h with 50 µM MG132 and then transduced with the indicated VLPs at MOI of 10. 24 h later, UNG2 content was analyzed with anti-UNG2 by western blotting (lanes were cropped from different parts of a unique gel (see Additional file 2: Supplementary dataset 2)) and UNG activity as in (**a**). Values are the means of triplicate measurements ± SD. Statistical significance was determined using the ANOVA test. Ut: untransduced

Viral stocks were produced by calcium phosphate transfection of HEK 293T cells. Briefly, cells were transfected with the proviral DNA constructs for 8 h, washed with pre-warmed DMEM-10% SVF medium. Viral supernatants were collected 48 h post-transfection, filtered and frozen in aliquots at − 80 °C. HA-Vpr delivering VLPs were purified by centrifugation through 20% sucrose cushion at 25,000 rpm for 2.5 h at 4 °C in a Sw32Ti rotor (Beckman Coulter). The pellets were resuspended in PBS, aliquoted and stored at − 80 °C. The Vpr content of purified VLPs was checked by western blot analysis **(**Fig. 2a**)**. Viral stocks were titered using HIV-1 p24 Enzyme-linked immunosorbent assay (ELISA) kit (Ingen) and focus forming assay (FFA) following the expression of GFP from the lentiviral construct (Figs. 1c and 2a). Viral stocks of *wt* or ΔVpr HIV-1 (NL4-3) were produced as described above in HEK 293T cells and titered as above. VSV-G pseudotyped *wt* HIV-1 viral stocks were produced using co-transfection of HEK 293T cells with a NL4-3 Δenv derivative and pCMV-VSV-G construct.

### Western blots

Proteins were separated by 12.5% SDS–PAGE gels, transferred to a PVDF membrane (Millipore) and immunoblotted with the appropriate primary and horseradish peroxidase–conjugated secondary antibodies. Immune complexes were revealed by Enhanced Chemiluminescence (Thermo Scientific) and recorded with the Gene Gnome imaging system (SynGene). Band intensities were quantified using ImageJ software.

### Uracil DNA glycosylase assays

The fluorescent probe PEG-U9 consisted of a 5′-FAM-GCACUUAAGAAUUG-PEG-CAAUUGUUAAGUGC-3′DAB oligonucleotide synthesized by Eurogentec [22]. Uracil DNA glycosylase activity was assayed in a reaction buffer containing 20 mM Tris–HCl pH 8.0, 50 mM KCl, 0.2 mM MgCl_2_, 0.05% Brij-35. The mixture was incubated with PEG-U9 (100 nM) for 30 min at 37 °C. Fluorescence was recorded using a Tecan Infinite 200 fluorimeter.

### Genome uracil content quantification

Genomic DNA was extracted from 3 × 10^6^ cells using DNAzol (Invitrogen) according to manufacturer’s recommendations. Pre-existing abasic sites (AP sites) in DNA were protected by incubation with methoxyamine (200 mM) for 2 h at 37 °C. Then 4 µg DNA was incubated in the presence of 0.2 U recombinant *E. coli* UDG (New England Biolabs) for 15 min at 37 °C. Apurinic/apyrimidinic sites (AP) generated by excision of uracil residues contained in DNA were titrated using the OxiSelect oxidative DNA Damage ELISA kit (Euromedex). Alternatively, genomic uracil content was determined by LC/MS/MS method [34]. Briefly, DNA was isolated by phenol:chloroform:isoamyl extraction, treated with alkaline phosphatase to remove free deoxyribonucleotides, and then enzymatically hydrolyzed to deoxyribonucleosides. Deoxyuridine (dU) was then separated from deoxycytidine (dC) by HPLC fractionation using a reverse-phase column with embedded weak acidic ion-pairing groups (2.1 mm × 150 mm, 5 μm, Primesep 200, SIELC technologies), using a water/acetonitrile gradient containing 0.1% formic acid. The dU fraction was finally analyzed by ESI-LC/MS/MS using a reverse phase column (2.1 mm × 150 mm, 3.5 µm, Zorbax SB-C18, Agilent Technologies), using a water/methanol gradient containing 0.1% formic acid on an API5000 triple quadrupole mass spectrometer (Applied Biosystems) in positive ionization mode.

### Class switch DNA recombination

CH12F3 cells were transduced with VLP HA-Vpr or ΔVpr. 24 h later, transduced cells were stimulated to undergo IgM-to-IgA switching with 5 ng/mL mouse recombinant IL4 (Peprotech), 200 ng/mL anti-CD40 monoclonal antibody (clone HM40-3, eBiosciences) and 1 ng/mL hTGF-β (R&D Systems) for 3 days. After labeling cells with anti-B220-APC (clone RA3-6B2, eBiosciences) and anti-IgA-PE (Southern Biotechnology), CSR efficiencies (% of IgGA^+^ B220^+^ cells) were evaluated by flow cytometry. Primary mature mouse B-cells were transduced with Vpr-delivering VLPs or control VLPs, and stimulated 24 h later to undergo IgM-to-IgG1 switching with 40 μg/mL LPS (Salmonella typhimurium, R&D Systems) and 50 ng/mL IL-4 (PeproTech) for 3 days. After labeling with anti B220-APC (clone RA3-6B2, eBiosciences) and anti-IgG1-PE (clone A85-1, BD Biosciences), CSR efficiencies (% of IgG1^+^ B220^+^ cells) were evaluated by flow cytometry. Data were acquired on a BD LSRII Fortessa cytometer and analyzed with the BD FACSDiva 6.1.3 software. Cell proliferation was measured with standard MTT/formazan colorimetric assay at 550 nm [35].

### Statistical analysis

Statistical analysis were performed using either ΣZanalyze software (http://www.ezanalyze.com; Poynton (2007) Version 3.0), Boston MA, USA) by one-way analysis of variance (ANOVA), or with GraphPad Prism 5.04 (GraphPad software, La Jolla USA).

## Results

### Production of Vpr-delivering virus-like pseudoparticles (VLPs)

As a prerequisite to this study, we first ensured that UNG2 expressed in human B-cells was susceptible to the Vpr-mediated depletion we previously identified in HIV-1 infected human T-cells [22]. The infection of Daudi B-cells with VSV-G pseudotyped *wt* HIV-1 showed clear UNG2 depletion that started 24 h.p.i with a 53% decrease in UNG2 levels and reached 96% UNG2 depletion at 72 h.p.i **(**Fig. 1a**)**. Similarly, infection of Daudi-CD4^+^ cells, permissive to HIV-1 infection [36], with *wt* HIV-1 or VSV-G pseudotyped *wt* HIV-1 generated UNG2 depletion reaching 48% and 92%, respectively (Fig. 1b). UNG2 levels were unchanged in cells infected with HIV-1 ΔVpr or in uninfected cells. These data therefore confirm the ability of HIV-1 Vpr to induce UNG2 depletion in cells of the B lineage.

In order to circumvent the well-known intrinsic limitations of producing functional recombinant Vpr versions [37], and to better control Vpr amounts delivered to B-cells, we generated VSV-G-pseudotyped virus-like HIV-1 particles (VLPs) able to incorporate HA-tagged Vpr by binding to the p6 domain of p55 Gag [38], referred to below as Vpr-delivering VLPs (VLP HA-Vpr) (Fig. 1c). VLPs lacking Vpr incorporation (VLP ΔVpr) were produced for control conditions. The purification of VLP HA-Vpr by sucrose gradient centrifugation confirmed the correct encapsidation of HA-Vpr in VLPs (Fig. 1c). We could approximate, from western blots performed on sucrose gradient-purified VLPs, the amount of VLP-encapsidated Vpr to be approximately 40 ng per µg of p24 corresponding to ≈ 760 molecules of Vpr *per* virion, close to the average number of Vpr found *per* HIV-1 mature particles, i.e. 700 molecules [39]. Daudi immortalized human B lymphocytes were transduced by both types of VLPs at MOI of 10 (Fig. 1d). In these conditions, UNG2 protein was decreased starting from 48 h post-transduction. A clear depletion (over 60% depletion) was reached 72 h later with Vpr-delivering VLPs while UNG2 levels remained unchanged in cells challenged with VLP ΔVpr. At later time points VLP HA-Vpr was clearly inducing total UNG2 depletion (Fig. 1d). In these conditions, Vpr could not be detected by western blot in cell extracts. Assuming a 100% transduction/entry yield, we can only expect a total Vpr amount of 460 femtograms from 3 × 10^6^ transduced cells, which is substantially below the detection limit of western blots. Altogether, these data therefore validated the ability of a very low amount of Vpr delivered by lentiviral vectors to efficiently induce the degradation of UNG2 in human B lymphocytes. This Vpr-delivery system therefore efficiently phenocopied Vpr functions with regards to UNG2 depletion and loss of uracil excision capacity previously observed in T lymphocytes [22].

### Vpr induces proteasome-dependent UNG2 degradation which decreases uracil excision capacity of immortalized human B-cells

Vpr function in B-cells was further challenged by the use of Vpr mutants with altered properties. Daudi cells were transduced for 72 h with VLPs delivering HA-tagged wild type Vpr (HA-Vpr *wt*) or Vpr mutants (R90K, Q65R and W54R) (Fig. 2a). UNG2 depletion was confirmed with Vpr *wt.* It was also detected to the same extent for the Vpr R90K mutant which was unable to induce G2/M cell cycle arrest [40] but still competent to interact with human UNG2 [9, 22] (Fig. 2a). On the other hand, Vpr mutant Q65R which does not recruit DCAF1 [41] and W54R which does not interact with UNG2 [42], left both UNG2 levels and UNG activity unchanged. Vpr-free VLPs (ΔVpr) were used as negative controls.

Several Vpr functions are dependent on the rerouting of host ubiquitination pathways [43, 44]. In epithelial cells [9, 45] and HIV-replicating T lymphocytes [22], Vpr has been shown to enhance UNG2 proteasome-dependent degradation [46], that relies on the ability of Vpr to recruit the DCAF1-Cul4A E3 ligase complex [8]. In our hands, treatment of Daudi cells with the proteasome inhibitor MG132 prior to transduction with HA-Vpr *wt*-delivering VLPs prevented Vpr-mediated UNG2 degradation and subsequent decreased UNG activity (Fig. 2b). These observations further established that Vpr-delivering VLPs reproduced Vpr properties previously depicted for HIV-1 susceptible cells in the context of replicating virus.

### Vpr induced-UNG2 depletion increases genome uracilation in human B lymphocytes

**Fig. 3:**
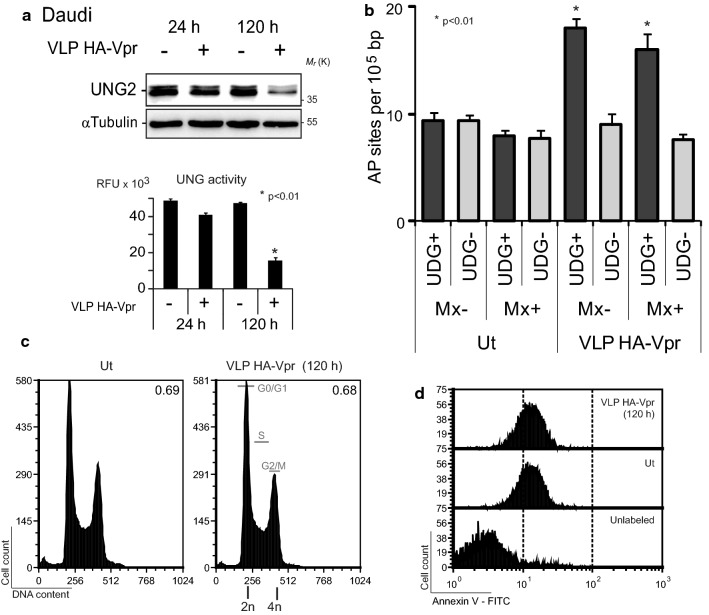
Transduction of Daudi B-cells by HA-Vpr-delivering VLPs increases genome uracilation. Daudi B-cells were transduced with VLP HA-Vpr at a MOI of 5. **a** UNG2 levels were analyzed by western blot with anti-UNG2 antibodies at 24 and 120 h post infection. Lanes were cropped from blot membranes serially probed with anti-UNG2 then with anti-αtubulin antibodies (see Additional file 2: Supplementary dataset 2). UNG activity was measured in the corresponding whole cell extracts. **b** DNA was extracted from cells 120 h post-transduction and treated with Methoxyamine (Mx) to protect pre-existing AP sites. Recombinant *E. coli* UDG was then used to excise uracil and generate AP sites which were quantified by ELISA. Numbers of AP sites per 10^5^ bp were measured in triplicate with the error bars representing the standard error of the mean. **c** DNA of ethanol-fixed cells was labeled with propidium iodide. Cell cycle progression was followed by flow cytometry. G2/M:G0/G1 ratios are indicated in the upper right corner of each histogram. **d** Apoptosis was followed by flow cytometry after labeling with Annexin V-FITC in the presence of CaCl_2_. Ut: untransduced

The lack of uracil excision in *ung*^−*/*−^ transgenic mice has been shown to induce massive genome uracilation and to dramatically increase the rate of spontaneous mutations in B-cells [47, 48] suggesting the absence of an efficient mechanism able to fully compensate for uracil excision deficiency. Vpr-mediated UNG2 depletion observed in Daudi B-cells (Fig. 1) can potentially give rise to an increase in genome uracilation. In order to test this consequence of UNG2 depletion, Daudi cells were transduced with VLP HA-Vpr using experimental conditions that induce significant UNG2 depletion (Fig. 3a). Five days after transduction, DNA was extracted and successively treated with methoxyamine (Mx) to protect endogenous or spontaneous apurinic/apyrimidinic (AP) sites, and with *E. coli* UDG to generate AP sites exclusively from uracil bases, and allow their further ELISA quantitation. Delivery of HA-Vpr to Daudi B-cells enhanced by two-fold the number of AP sites when compared to that of untransduced cells (Fig. 3b). The absolute AP site number specifically corresponded to uracil bases left unrepaired due to lack of UNG2 activity since AP site numbers only increased in UDG-treated samples (UDG^+^) and could not emerge from nonspecific or pre-existing AP sites (Mx^−^/UDG^−^ samples). In parallel, propidium iodide DNA staining and Annexin V labeling attested to the absence of G2/M cell cycle arrest (Fig. 3c) and apoptosis (Fig. 3d) in the corresponding cells. Consequently, the delivery of HA-Vpr to Daudi B-cells generates a noticeable UNG2 depletion that leads to a significant increase in genome uracilation in the absence of cell death or cell cycle inhibition.

### Vpr-induced loss of UNG2 activity correlates with CSR decline in stimulated immortalized mouse CH12F3 B-cells

The functional importance of UNG2 in B cell functions was first reported in *ung*^−*/*−^ mice [47, 48]. Besides resulting in a 100-fold increased steady-state uracil level in DNA associated with a non-negligible mutation rate increase (~ 1.5-fold), invalidation of the *ung* gene in these animals has major consequences on B-cells which can no longer undergo antibody class switch recombination and somatic hypermutation, resulting in significant reduction in IgG2b and IgG3 and alteration of IgM^+^ B-cells in germinal centers (GCs) [49, 50]. These animals also exhibit altered Ig responses to vaccine antigens or viral infections [49, 51]. Considering the requirement for UNG2 in antibody diversification which takes place in murine B-cells, the Vpr-driven deficit of UNG2 activity in these cells might greatly alter CSR and/or SHM efficiencies. In an attempt to challenge this hypothesis, we explored the impact of Vpr on the mouse CSR model cell line CH12F3 [52], which, in contrast with the Daudi cell line, has been widely used to accurately measure CSR efficacy and to identify CSR factors [53, 54].

**Fig. 4:**
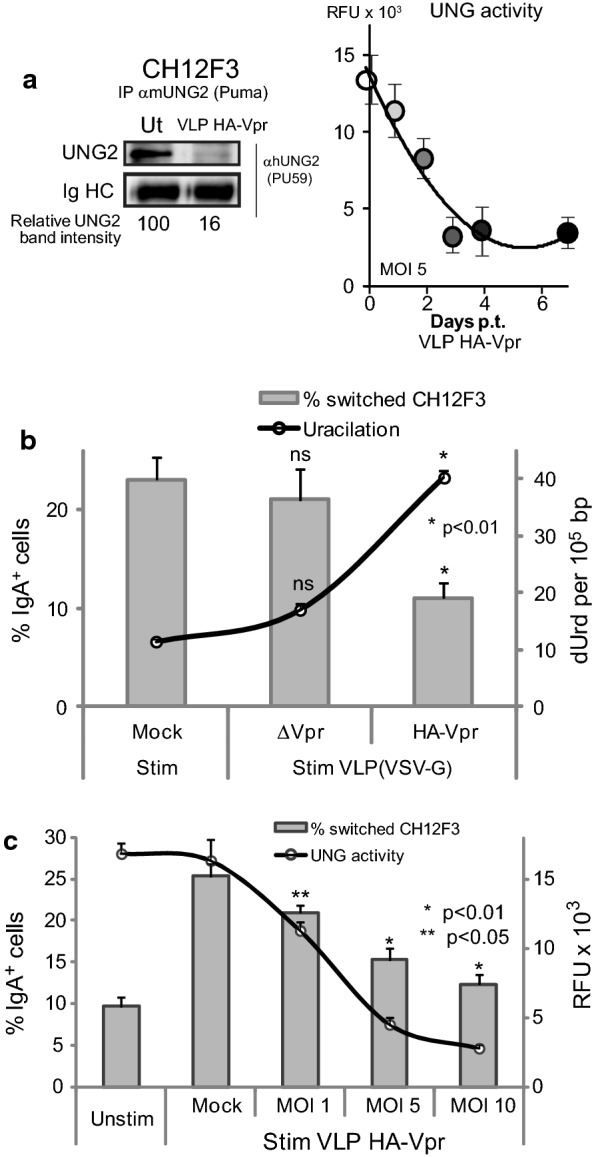
Impact of Vpr on CSR in immortalized B-cells. **a** *Left panel*, CH12F3 cells were transduced with VLP HA-Vpr at a M.O.I. of 10. 48 h later, UNG2 content was assessed by western blotting with anti-UNG2 antibodies as described in Materials and methods. Lanes were cropped from blot membranes serially probed with anti-UNG2 then with anti-αtubulin antibodies (see Additional file 2: Supplementary dataset 2). *Right panel*, CH12F3 cells transduced with VLP HA-Vpr (M.O.I. of 5) were examined for UNG activity at days 0, 1, 2, 3, 4 and 7. **b** After 24 h in culture CSR was induced by IL-4/α-CD40/TGF-β stimulation for 3 days. Class switch efficiency from IgM-to-IgA isotype was evaluated by flow cytometry by measuring the  % IgGA^+^ B220^+^ cells (left panel). DNA from corresponding cells was extracted and uracil content (dUrd per 10^5^ bp) was determined by LC/MS/MS. Cells transduced with VLP ΔVpr and mock-transduced cells are shown as controls. **c** CH12F3 B-cells were transduced with increasing MOI of VLP HA-Vpr. 24 h later, corresponding isotype switching was induced by cytokine stimulation for 3 days and evaluated by flow cytometry. Values are the mean of duplicate experiments ± SD. Corresponding UNG activities were measured in the presence or absence of the UNG inhibitor UGI. Stim: stimulated; Unstim: unstimulated

To this end, CH12F3 B-cells, previously transduced by VLP HA-Vpr or ΔVpr, were stimulated with anti-CD40 monoclonal antibody and hTGF-β for 3 days. Vpr delivery in these cells was controlled to reproduce the UNG2 outcome observed in human B-cells, namely complete depletion of UNG2 and decreased MOI-dependent UNG2 activity lasting for 4 to 6 days (Fig. 4a and c). Using these conditions, IgM-to-IgA CSR in HA-Vpr^+^ CH12F3 cells was clearly reduced with a concomitant increase in DNA uracilation (Fig. 4b), while they remained unchanged following transduction with VLP ΔVpr. In addition, IgM-to-IgA switch efficiency was only inhibited with native VLPs. Inhibition was no longer observed after heat inactivation of VLP HA-Vpr preparations (Additional file 1: Supplementary dataset 1) emphasizing that the observed inhibition was strictly dependent on accurate delivery of HA-Vpr and could not be ascribed to cytokine carry-over contaminations released by VLP-producing cells. Finally, since the intensity of UNG2 decrease was shown to be directly related to the amount of VLP used to transduce cells (Fig. 4c), the same correlation should be expected between VLP HA-Vpr load and CSR inhibition. The transduction of stimulated CH12F3 B-cells with increasing M.O.I. of HA-Vpr-delivering VLPs produced a dose-dependent decrease in CSR (Fig. 4c).

### IgG1 CSR proficiency of stimulated primary B-cells following transduction with HA-Vpr-delivering VLPs

**Fig. 5:**
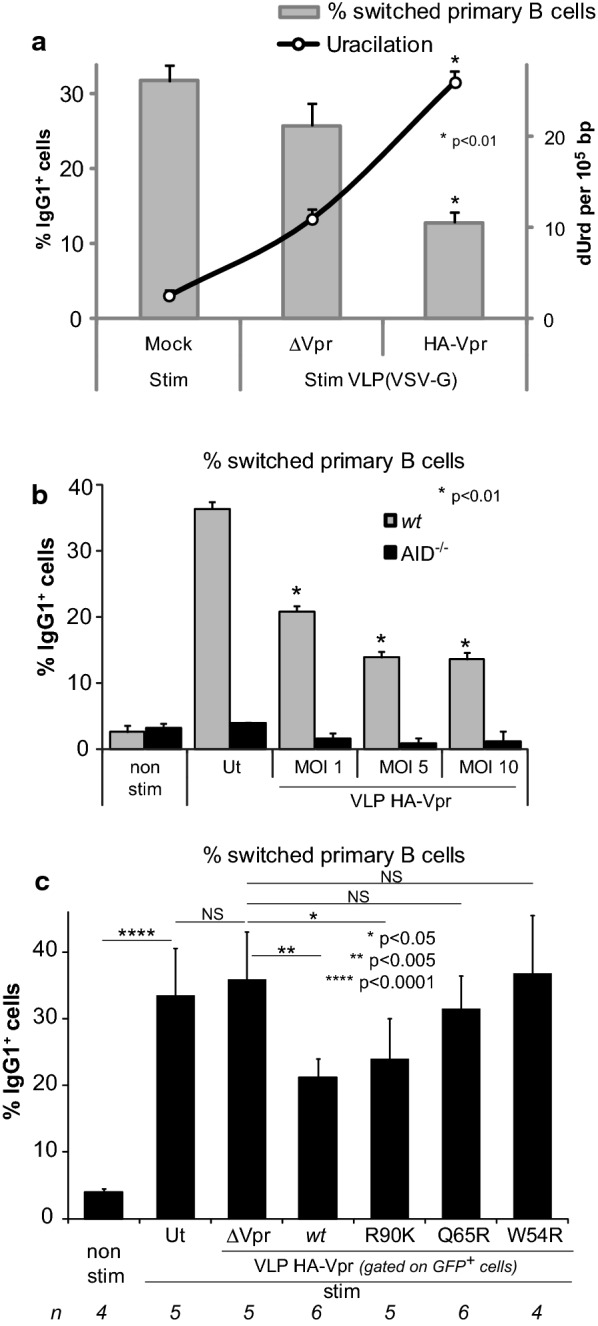
Impact of VLP-delivered Vpr on CSR efficiency in primary B-cells. **a** Primary B-cells were transduced with VLPs HA-Vpr or ΔVpr at a M.O.I. of 10. 24 h later CSR was induced by IL-4/α-CD40/TGF-β stimulation for 3 days. IgM-to-IgG1 isotype class switch efficiency was evaluated by flow cytometry by measuring the % IgG1^+^ B220^+^. DNA from corresponding cells was extracted and uracil content (dUrd per 10^5^ bp) was determined by LC/MS/MS. **b** Primary B-cells isolated from either *wt* C57BL/6 or AID^−/−^ mice, were transduced with increasing MOI of VLP HA-Vpr. 24 h later, isotype switching was induced by cytokine stimulation for 3 days and evaluated by flow cytometry. Values are the mean of duplicate experiments ± SD. Ut, untransduced, non stim: non stimulated. **c** Primary B-cells were transduced with VLP HA-Vpr (*wt* or mutants R90K, Q65R, W54R) or ΔVpr. 24 h later CSR was induced as stated above. Class switch efficiency was evaluated by flow cytometry by measuring % IgG1^+^ B220^+^ cells. For VLP-transduced cells, % switched cells was determined from GFP^+^-gated populations. Values are derived from three independent experiments with *n* corresponding to the number of replicates

Since the CH12F3 B-cell line is mostly committed to IgA isotype switching [52], which mainly occurs within intestinal mucosa in response to intestinal toxins and microbial pathogens [55], we used primary CD43^−^ mature B-cells isolated from *wt* C57BL/6 mice spleens that undergo IgG isotype switching and would be more relevant to evaluate the potential effects of Vpr on GC B-cells. When primary cells were transduced with VLP HA-Vpr, IgM-to-IgG1 CSR in HA-Vpr^+^ cells was reduced by half with a simultaneous twofold increase in genome uracilation (Fig. 5a) without altering cell proliferation (data not shown). As a control, in primary B-cells previously transduced by VLP HA-Vpr (native or heat inactivated (HI)), and then stimulated for 3 days with IL-4 and LPS, IgM-to-IgG1 switching was efficiently inhibited with native VLPs, and remained unchanged when cells were transduced with heat inactivated VLPs (Additional file 1: Supplementary data set 1). A dose-dependent decrease in IgG1 isotype switching was also observed when primary B-cells were transduced with increasing M.O.I. of HA-Vpr-delivering VLPs (Fig. 5b). In parallel, primary CD43^−^ mature B-cells from AID^−/−^ mice were used as a CSR negative control.

In order to confirm the connection between the observed Vpr-mediated CSR decrease and UNG2 depletion, we followed the CSR status of primary B-cells transduced by VLPs delivering Vpr mutants previously found unable to promote UNG2 degradation in Daudi B-cells (Fig. 2a). Both Vpr mutants (Q65R and W54R) did not alter CSR efficiency although Vpr *wt* and to a lower extent the R90K mutant, still reduced CSR from IgM-to-IgG1 (Fig. 5c). This observation clearly establishes that the observed Vpr-mediated effects on CSR can be ascribed to a deficiency in UNG activity.

We can therefore conclude that Vpr-mediated manipulation of uracil removal in B-cells impairs antibody diversification by altering class switch DNA recombination from C_µ_ to C_α_ and C_γ_.

### Vpr released from producing cells efficiently accumulates in a bystander B cell line

**Fig. 6:**
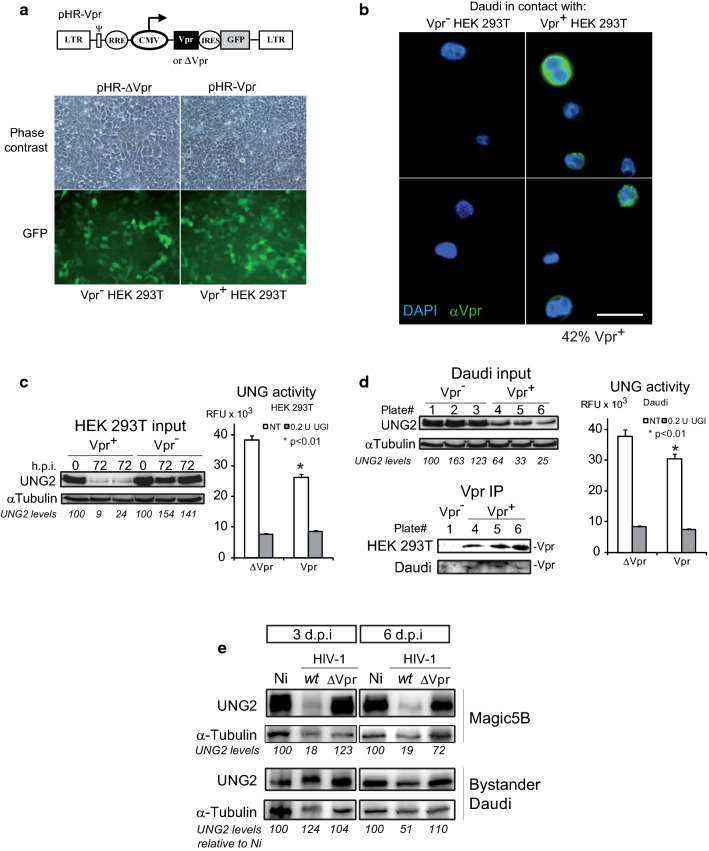
Vpr released from HEK 293T producing cells and Magic5B HIV-1 infected cells can be taken up by Daudi B-cells. **a** VLPs able to drive the constitutive expression of Vpr by delivery of the pHR-Vpr (Vpr^+^) lentiviral expression cassette or control VLPs containing a pHR-ΔVpr cassette were used to transduce HEK 293T. After 72 h, transduction efficiency was confirmed by the expression of GFP. **b** Cells were then washed and co-cultured with Daudi B-cells suspended in culture hanging inserts. After an additional 72 h, Daudi cells were collected, immobilized on poly(l-lysine) coated cover slips, formalin-fixed and labeled with DAPI and anti-Vpr antibodies to visualize nuclei and Vpr content, respectively. For each condition, whole cell extracts of producer HEK 293T cells (**c**) and of target Daudi cells (**d**) were analyzed for UNG2 content by immunoblot using αtubulin as a loading control. Lanes were cropped from blot membranes serially probed with anti-UNG2 then with anti-αTubulin antibodies (see Supplementary dataset). Uracil DNA glycosylase activity in the lysate was determined in the absence (NT) or presence of the UNG inhibitor UGI (0.2 U). Values are the means of triplicate experiments ± SD. Statistical significance was determined using the ANOVA test. Vpr content was determined by immunoprecipitation using anti-Vpr antibodies. Vpr was visualized by extending exposition time as specified in Additional file 2: Supplementary dataset 2. **e** Daudi cells maintained in hanging cell inserts were left in contact for the indicated time with MAGIC5B cells previously infected with HIV-1 *wt* or ΔVpr at MOI of 10. Target Daudi and producer MAGICB cell populations were harvested and whole cell extracts were prepared for immunoblot analysis of their UNG2 levels using αtubulin as a loading control. Lanes were cropped from blot membranes serially probed with anti-UNG2 then with anti-αTubulin antibodies (see Additional file 2: Supplementary dataset 2)

B lymphocytes are not permissive to HIV infection. Nevertheless, besides its presence in infected cells and in virions [56], Vpr was reported to exhibit an extracellular phase in sera and cerebro-spinal fluid from infected patients [29] with concentrations ranging from pM to nM in serum [57]. Vpr is endowed with protein-transducing properties [27] that allow it to penetrate a broad array of cells [28]. Detection of Vpr accumulated in GC B-cells of HIV-infected patients [58] further supports this aptitude. Nevertheless, the effective occurrence of cell-free Vpr and its capacity to penetrate bystander cells still remain a matter of debate [59–61]. In order to test whether Vpr can actually be secreted and thereafter taken up by bystander human B-cells to manipulate UNG2, we made use of a co-culture format between HEK 293T cells transduced with the pHR-Vpr construct to constitutively express Vpr protein (Fig. 6a) and bystander Daudi B-cells. After a 3 day production period in HEK 293T cells visualized by GFP expression (Fig. 6a), and the expected UNG2 depletion and decreased uracil removal activity (Fig. 6c), Daudi B-cells were brought in contact in hanging cell inserts. After an additional 3 days, the actual intercellular transfer of Vpr was visualized by immunofluorescence staining of Daudi cells with anti-Vpr antibodies (Fig. 6b). Vpr noticeably accumulated in more than 42% of exposed cells. Concurrently, Vpr was also detected by western blotting of the corresponding whole cell extracts after Vpr immunoprecipitation (Fig. 6d). Vpr accumulation was concomitant with a noticeable decrease in UNG2 observed on western blots of whole Daudi cell extracts and with a significant reduction in UNG activity (Fig. 6d). None of these effects was observed when Daudi cells were co-cultured with non-producing HEK 293T cells. In order to confirm that Vpr released from HIV-1 infected cells could still deregulate UNG2 in bystander B-cells, Daudi cells were left in contact in hanging cell inserts with MAGIC5B cells previously infected by either *wt* or ΔVpr HIV-1. After 6 days contact, UNG2 levels were decreased by 51% in Daudi cells in contact with producer cells infected with *wt* HIV-1 (Fig. 6e), whereas they remained unchanged at shorter time points (3 days) or when producer cells were either infected by ΔVpr HIV-1 or mock-infected. Taken together, these data clearly demonstrate the ability of Vpr released from producing cells and from HIV-1 infected cells to accumulate and manipulate UNG2 activity in bystander human B lymphocytes.

## Discussion

UNG2 has a significant impact on uracil control and therefore in maintaining genome integrity in all cell types. In addition it also displays a mutagenic role in B-cells where it is involved in CSR and SHM at the immunoglobulin (Ig) heavy chain (*IGH)* locus [62]. CSR is initiated by antigen stimulation and induction of the cytidine deaminase AID [63] which deaminates cytosine residues in the single stranded DNA present in transcriptional loops of the variable (V) and switch (S) regions of the *IGH* genes to generate the accumulation of uracil bases. Excision of these bases by UNG2 forms abasic sites (AP sites) cleaved by APE1. When this process occurs on both DNA strands, formation of double-strand breaks initiates a recombination process that leads to the switch of Ig heavy chain constant gene segments [64]. The joint action of AID and UNG2 is also necessary to accomplish the mutagenic processing of variable gene segments by somatic hypermutation (SHM) leading to increased affinity of antibodies for their target [65].

We have previously shown that Vpr expression in the context of HIV-1 infection markedly decreases UNG2 expression in transformed or primary CD4 + T lymphocytes, and demonstrated for the first time that Vpr-UNG2 interaction significantly impairs the uracil excision activity of these infected cells [22]. The data presented herein are the first to confirm the capacity of low amounts of Vpr to deregulate UNG2 in cells of the B lymphoid lineage. Using a VLP-based delivery approach able to mimic free Vpr addressing, we found that Vpr delivery to bystander B-cells efficiently phenocopies proteasome-dependent UNG2 degradation previously reported in T lymphocytes and epithelial cells. These effects were independent of Vpr cytostatic properties. In immortalized and primary mouse B-cells, Vpr induced a dose-dependent loss of control of overall genome uracilation, and predominantly interrupted AID-dependent generation of AP sites required for CSR completion in activated B lymphocytes [66]. HA-Vpr delivering VLPs fine tuned UNG activity decrease which was compatible with the cytokine stimulation time required to induce CSR in these cells. Using these conditions, we observed CSR inhibition rates reaching 44% and 58%, in immortalized and primary B-cells, respectively.

After showing that Vpr delivered by transducing VLPs may manipulate UNG2-dependent CSR, we re-examined the ability of Vpr to be released by producing cells and to effectively trigger Vpr accumulation in a B-cell line. For over two decades, the existence of an extracellular stage of HIV proteins has been the subject of intense controversy. Auxiliary proteins such as Tat, Nef and Vpr were reported to be produced in infected patients and as a result of low noise transcription of HIV-1 mRNA despite efficient blockade of virion production by cART [67, 68]. Once secreted, these proteins were proposed to accumulate in various tissues including lymphoid tissues, mainly within lymph node germinal centers, where they may act as critical micro-environmental factors [69]. Here, we developed a co-culture system between Vpr-producing cells and bystander B-cells maintained in hanging cell inserts. We found that Vpr produced from epithelial cells was able to penetrate B-cell membranes and downregulate UNG2 expression and associated enzyme functions. This effect reproduced those recorded using Vpr-delivering VLPs. In this system, the overall Vpr amount present in the VLPs in contact with B-cells was estimated to correspond to a free Vpr concentration in the femtomolar range thereby mimicking picomolar to nanomolar Vpr concentrations detected in the body fluids of HIV-1 patients [59, 70, 71]. Finally, Vpr-mediated UNG2 deregulation was also confirmed using recombinant GST-Vpr produced in *E. coli* (data not shown). The high toxicity GST-Vpr was inducing, in part due to the GST moiety, considerably limited its further utilization.

Altogether our observations broaden the extensive exploration of the physiopathological consequences circulating Vpr may have. Indeed, cell-free Vpr has been proposed to contribute to HIV-associated lipodystrophy (HAL) [70], AIDS cardiomyopathy (AIDS-CM) [72] and HIV-associated neurological disorders (HAND) [73–75] thereby potentially causing AIDS-associated syndromes despite the efficacy of highly active anti-retroviral therapy [76]. Our results raise questions as to whether Vpr participates in HIV-associated humoral immunity dysfunction. In HIV-1 infected patients, B-cell dysfunctions mainly originate from activation-induced B-cell exhaustion and loss of CD4^+^ T-cells, which are absolutely required to assist follicular B-cells via CD40 ligand (CD40L)-CD40 interaction [77]. However, normalization of CD4^+^ T cells by cART, while able to re-establish the proportion of different B-cells subpopulations in chronically infected patients [78], does not fully restore antigen-specific IgG and IgA responses [79] pointing to the likelihood of intrinsic memory B-cell abnormalities [80, 81]. In perinatally HIV-infected children, both HIV-seropositive and seronegative patients were identified among those that had received early cART therapy even if similar numbers of gp140-specific B- and T-cells were found in the two groups [82]. While the mechanisms accounting for such intrinsic defects remain poorly defined, a higher baseline of AID expression in B-cells was mainly credited to the CD27^−^ B-cells subpopulation in HIV-1 patients compared to healthy controls [83]. Moreover, although normally limited to GC B-cells [84], high levels of extra-follicular AID expression occur in non-stimulated PBMCs from HIV-1 infected patients. Together with our observations this suggests that SHM and CSR impairments could be related to a loss of specificity of AID-induced mutations which is known to be resolved by the essential presence of UNG2 [65]. It is commonly admitted that HIV-associated B-cell intrinsic defects can result from the intercellular trafficking of several viral accessory proteins whose expression levels remain detectable under cART therapy [57]. Via its penetration in myeloid cells, Nef can suppress CD40-dependent immunoglobulin class switching in bystander B-cells [85] and virus-specific IgG2 and IgA class switching [86]. Vif accumulates in GCs and directly inhibits the enzyme activity of B-cell specific AID [87] Recently, using an approach very similar to ours, circulating Tat protein was shown to enter human B-cells subsequently inducing AID-dependent somatic hypermutations (SHM) that could contribute to the increased lymphomagenesis in ART-treated HIV-positive population [88].

Overall, our results highlight the ability of virus-free/cell-free HIV-1 Vpr to penetrate and to manipulate UNG2 in bystander B-cells. They reveal an unexpected capability of VLPs-delivered Vpr to interfere with CSR processes in B lymphocytes through the proteasome-based manipulation of the UNG2 DNA repair enzyme. Meanwhile, the Vpr delivering tools we have developed might be used reliably to facilitate the investigation of CSR and SHM mechanisms by specifically altering UNG2 contribution in human B-cells. Our results raise the question of the pathogenic consequences of Vpr-induced UNG2 depletion and CSR deregulation that we observed in B lymphocytes. Although debated, the widespread occurrence of cell-free Vpr, the capacity of Vpr to penetrate bystander cells and its detection in B-cells present in germinal centers in HIV-infected patients [58], together with Vpr-mediated CSR deregulation reported herein, suggest that Vpr potentially contributes to HIV-associated B-cell intrinsic defects, to the loss of class-switched memory B-cells observed in both viremic and aviremic patients [79, 89] and possibly to the lower humoral response against circulating antigens [82, 90] or in response to vaccination [91, 92] reported in HIV-infected children and adults. Further studies are therefore needed to clearly evaluate the precise contribution of cell-free Vpr on B-cells functions in HIV-infected individuals. This will especially benefit recently from developed quantitative systems for monitoring blood‐circulating Vpr in relation to pathogenic indices [93].

## Conclusion

The here described potential evasion of HIV-1 from antibody class switching by the Vpr-mediated UNG2 degradation in bystander B-cells, paves the way towards a better understanding of HIV-related humoral deficiencies and suggests the ablation of Vpr coding sequences from vaccine strategies. In addition, considering the extensive proteome remodeling Vpr can induce in infected cells [94] and various bystander cells, as well as pharmacological strategies aimed to interfere with its multiple functions [95, 96], Vpr has re-emerged as an attractive target for HIV disease treatment [97].

## Supplementary information

**Additional file 1.** Supplementary dataset 1. Supplementary Figure S1. (a) CH12F3 cells were transduced with native or 30 min heat inactivated (56°C) VLP HA-Vpr at MOI of 5. CH12F3 cell switching from IgM to IgA isotype was induced 24 hours later by IL-4/α-CD40/TGF-β stimulation for 3 days. CSR efficiencies were evaluated by flow cytometry by measuring the % of IgGA^+^ B220^+^ cells. (b) Cell proliferation was evaluated by a MTT/formazan cell viability colorimetric assay (right panel). (c) Mouse primary B-cells were transduced with native or 30 min heat inactivated (56°C) HA-Vpr at MOI of 5. IgM to IgG1 isotype switching was induced by LPS/IL-4 stimulation for 3 days. (d) Cell proliferation was evaluated as in (b).

**Additional file 2.** Supplementary dataset 2. Original western blot including cropping and exposure strength strategies.

## Data Availability

The Additional file 1: Supplementary dataset 1 contains supplementary Figure S1 that describes additional control class switch recombination experiments. Additional file 2: Supplementary dataset 2 contains gel source data (original western blots) with cropping and exposure strength strategies.
