# Supplementary material for: Impact of HIV-1 Vpr manipulation of the DNA repair enzyme UNG2 on B lymphocyte class switch recombination
Source: J Transl Med. 2020 Aug 10;18:310. doi: 10.1186/s12967-020-02478-7 (PMC7418440; doi:10.1186/s12967-020-02478-7)
Supplement: Supplementary file 1 — Additional file 1. Supplementary dataset 1. Supplementary Figure S1. (a) CH12F3 cells were transduced with native or 30 min heat inactivated (56°C) VLP HA-Vpr at MOI of 5. CH12F3 cell switching from IgM to IgA isotype was induced 24 hours later by IL-4/α-CD40/TGF-β stimulation for 3 days. CSR efficiencies were evaluated by flow cytometry by measuring the % of IgGA+ B220+ cells. (b) Cell proliferation was evaluated by a MTT/formazan cell viability colorimetric assay (right panel). (c) Mouse primary B-cells were transduced with native or 30 min heat inactivated (56°C) HA-Vpr at MOI of 5. IgM to IgG1 isotype switching was induced by LPS/IL-4 stimulation for 3 days. (d) Cell proliferation was evaluated as in (b). [file 12967_2020_2478_MOESM1_ESM.pdf]

Impact of HIV-1 Vpr manipulation of the DNA repair enzyme UNG2 on B lymphocyte class switch recombination

Patrick Eldin, Sophie Péron, Anastasia Galashevskaya, Nicolas Denis-Lagache, Michel Cogné, Geir Slupphaug and Laurence Briant

Supplementary Dataset 1

Supplementary Figure-S1

Supplementary Figure-S1 (eldin)

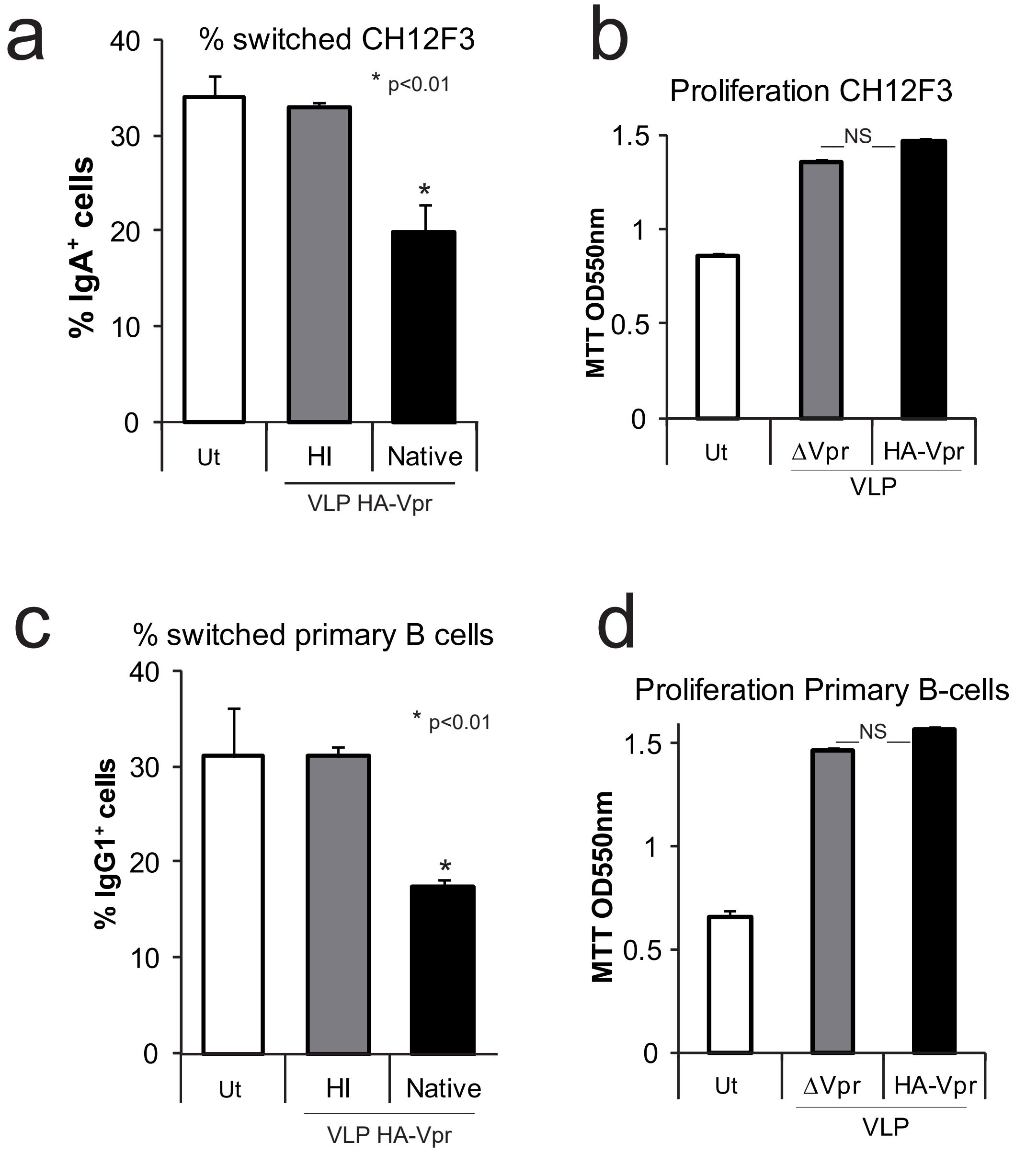

(a) CH12F3 cells were transduced with native or 30 min heat inactivated (56°C) VLP HA-Vpr at MOI of 5. CH12F3 cell switching from IgM to IgA isotype was induced 24 hours later by IL-4/α-CD40/TGF-β stimulation for 3 days. CSR efficiencies were evaluated by flow cytometry by measuring the % of IgGA$^+$B220$^+$ cells. **(b)** Cell proliferation was evaluated by a MTT/formazan cell viability colorimetric assay (right panel). **(c)** Mouse primary B-cells were transduced with native or 30 min heat inactivated (56°C) HA-Vpr at MOI of 5. IgM to IgG1 isotype switching was induced by LPS/IL-4 stimulation for 3 days. **(d)** Cell proliferation was evaluated as in (b).
