# Supplementary material for: Impact of HIV-1 Vpr manipulation of the DNA repair enzyme UNG2 on B lymphocyte class switch recombination
Source: J Transl Med. 2020 Aug 10;18:310. doi: 10.1186/s12967-020-02478-7 (PMC7418440; doi:10.1186/s12967-020-02478-7)
Supplement: Supplementary file 2 — Additional file 2. Supplementary dataset 2. Original western blot including cropping and exposure strength strategies. [file 12967_2020_2478_MOESM2_ESM.pdf]

Impact of HIV-1 Vpr manipulation of the DNA repair enzyme UNG2 on B lymphocyte class switch recombination

Patrick Eldin, Sophie Péron, Anastasia Galashevskaya, Nicolas Denis-Lagache, Michel Cogné, Geir Slupphaug and Laurence Briant

Supplementary Dataset 2

Original western blot including cropping and exposure strength strategies

Daudi

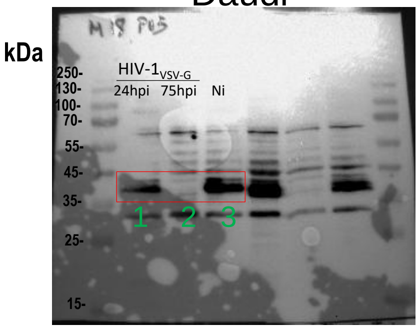

Anti-UNG2 luminescence merged with colorimetric

FIG 1a

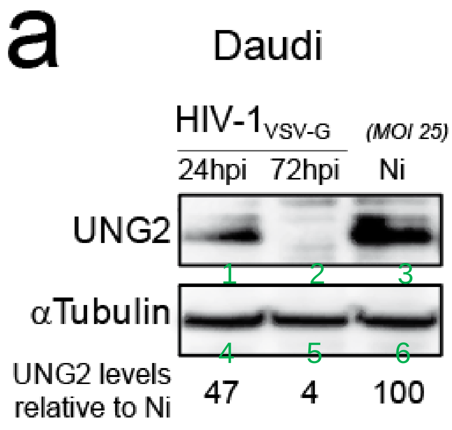

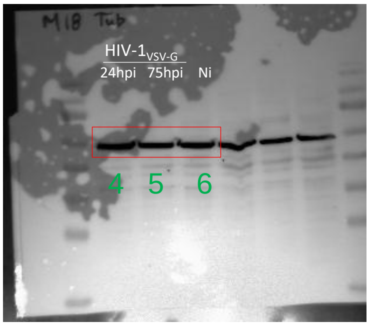

Anti-Tubulin luminescence merged with colorimetric (parallel gel)

Cropped area

Lane numbers

Daudi-CD4+

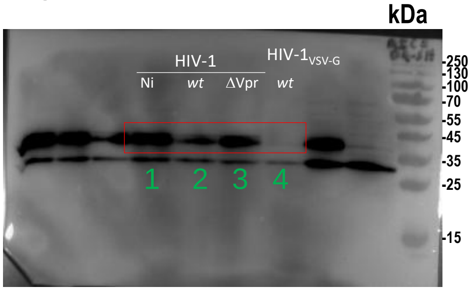

1st probing: Anti-UNG2 luminescence merged with colorimetric

FIG 1b

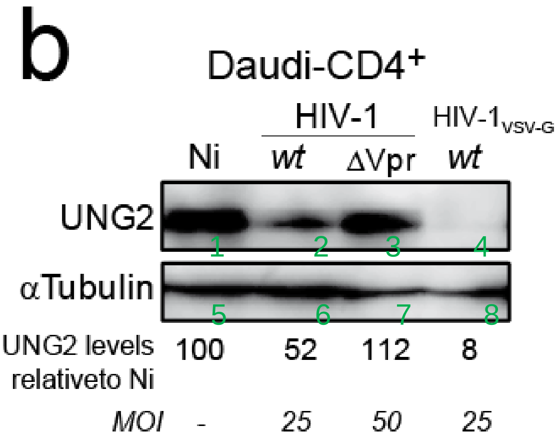

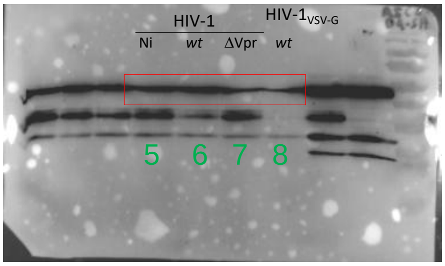

2nd Probing: Anti-Tubulin luminescence merged with colorimetric

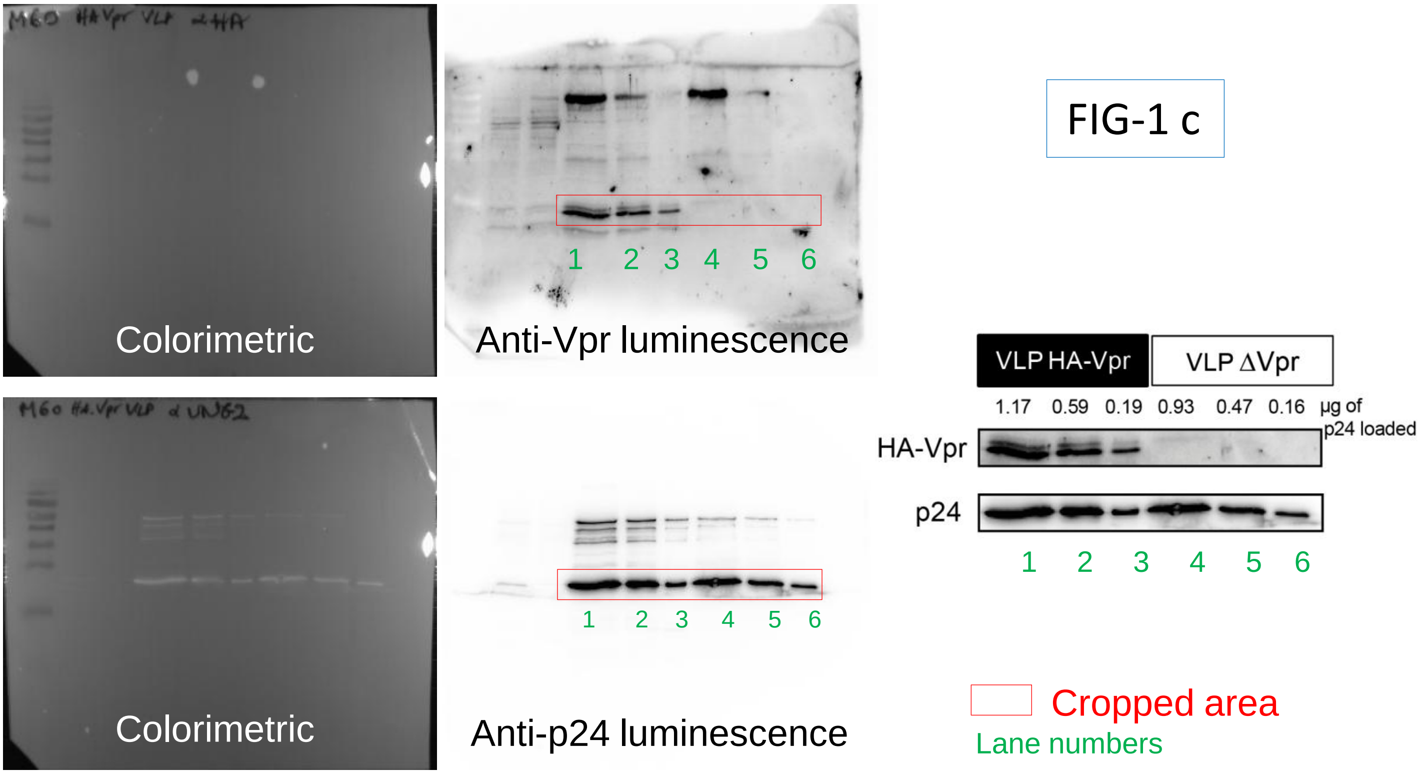
FIG-1 c
Colorimetric
Anti-Vpr luminescence
Colorimetric
Anti-p24 luminescence
1 2 3 4 5 6
VLP HA-Vpr
VLP ΔVpr
1.17 0.59 0.19 0.93 0.47 0.16 μg of p24 loaded
HA-Vpr
p24
Cropped area
Lane numbers

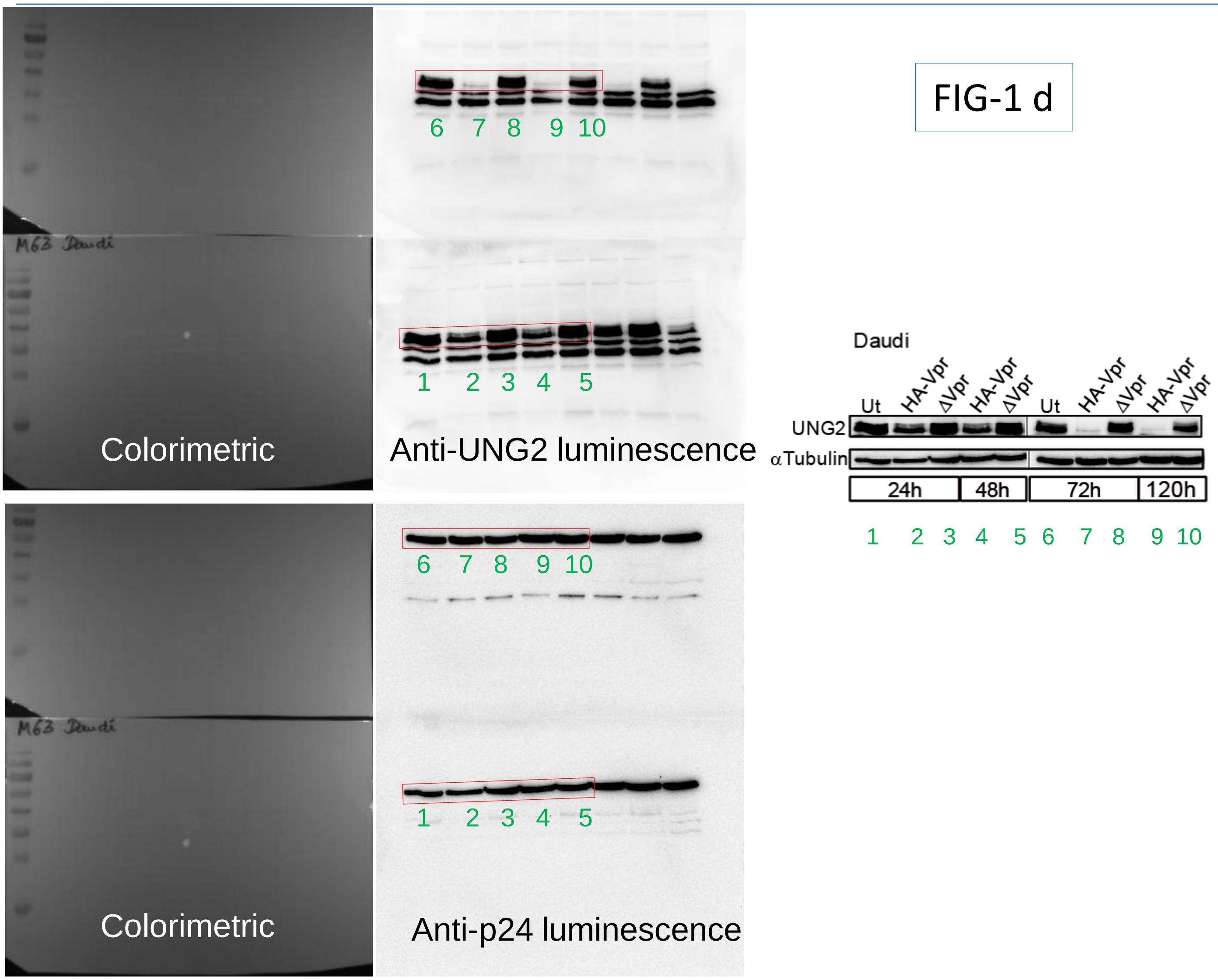
FIG-1 d
6 7 8 9 10
1 2 3 4 5
Colorimetric
Anti-UNG2 luminescence
Daudi
Ut HA-Vpr ΔVpr HA-Vpr ΔVpr Ut HA-Vpr ΔVpr HA-Vpr ΔVpr
UNG2
αTubulin
24h 48h 72h 120h
1 2 3 4 5 6 7 8 9 10
Colorimetric
Anti-p24 luminescence

FIG-2 a

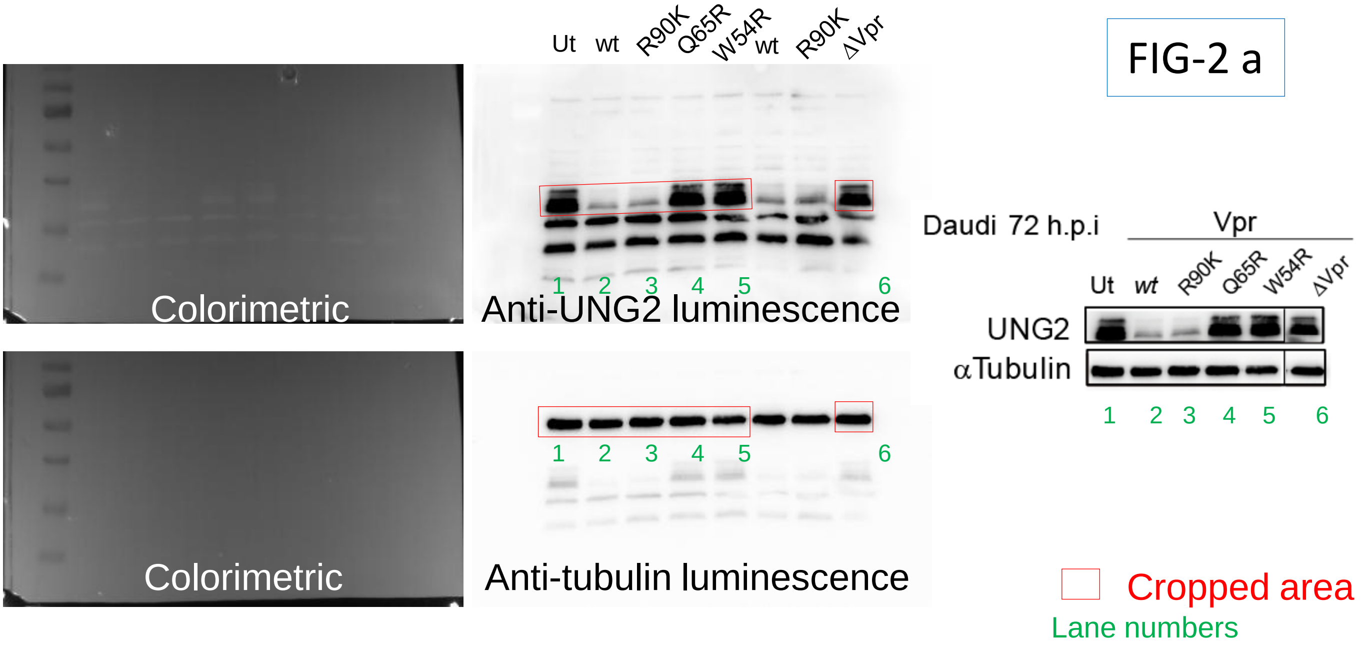
Ut wt R90K Q65R W54R wt R90K ΔVpr
Colorimetric
Anti-UNG2 luminescence
1 2 3 4 5 6
Colorimetric
Anti-tubulin luminescence
1 2 3 4 5 6
Daudi 72 h.p.i
Vpr
Ut wt R90K Q65R W54R ΔVpr
UNG2
αTubulin
1 2 3 4 5 6
Cropped area
Lane numbers

FIG-2 b

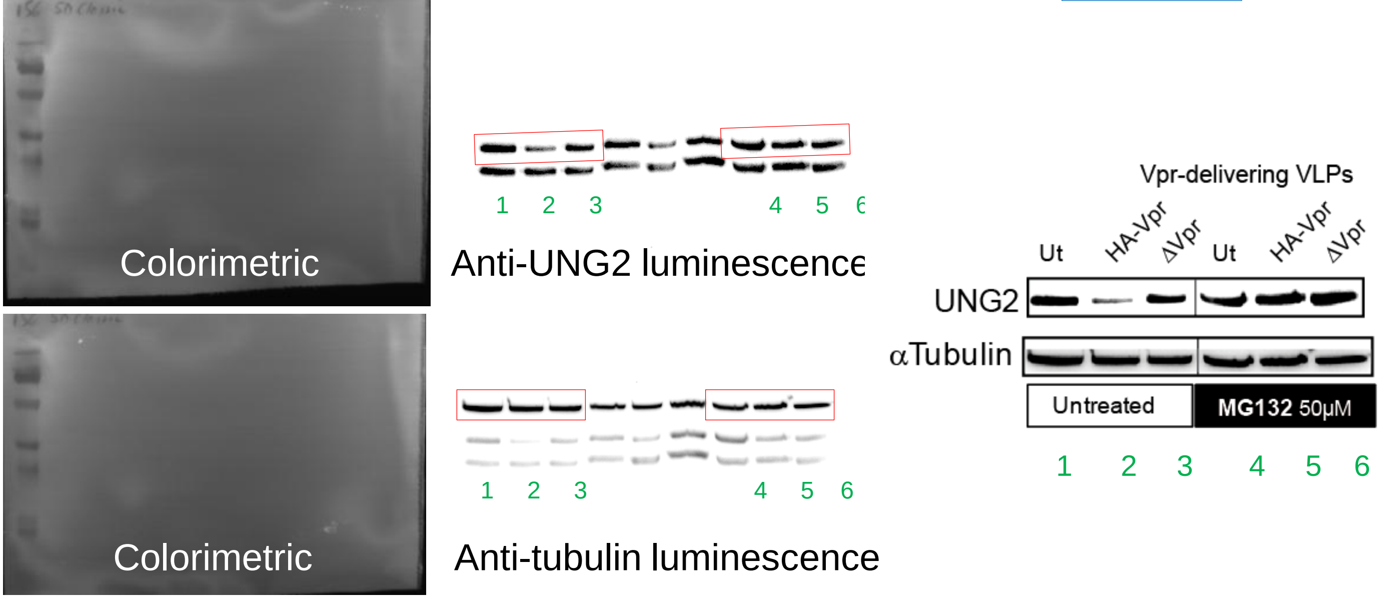
Colorimetric
Anti-UNG2 luminescence
1 2 3 4 5 6
Colorimetric
Anti-tubulin luminescence
1 2 3 4 5 6
Vpr-delivering VLPs
Ut HA-Vpr ΔVpr Ut HA-Vpr ΔVpr
UNG2
αTubulin
Untreated
MG132 50μM
1 2 3 4 5 6

FIG-3

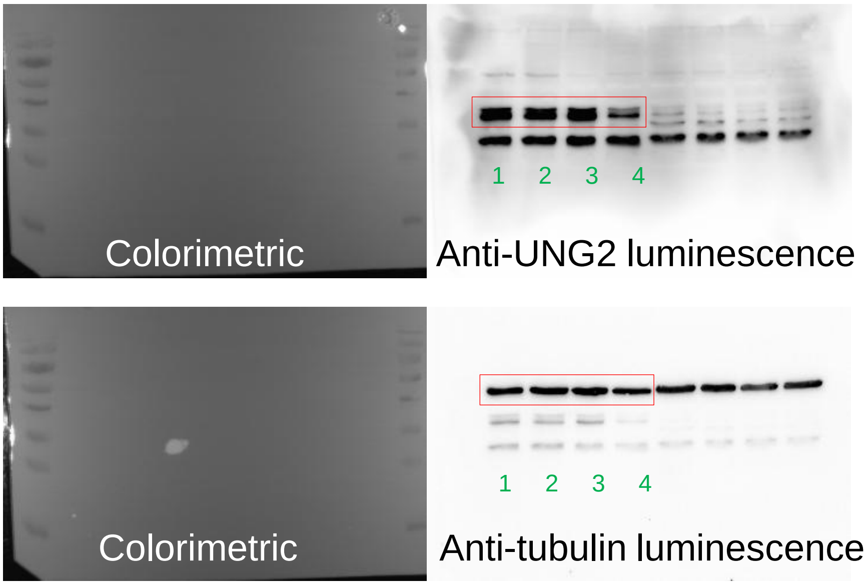

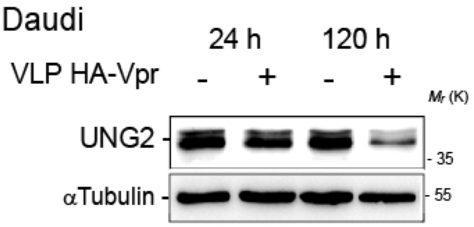

Cropped area

Lane numbers

FIG-4a

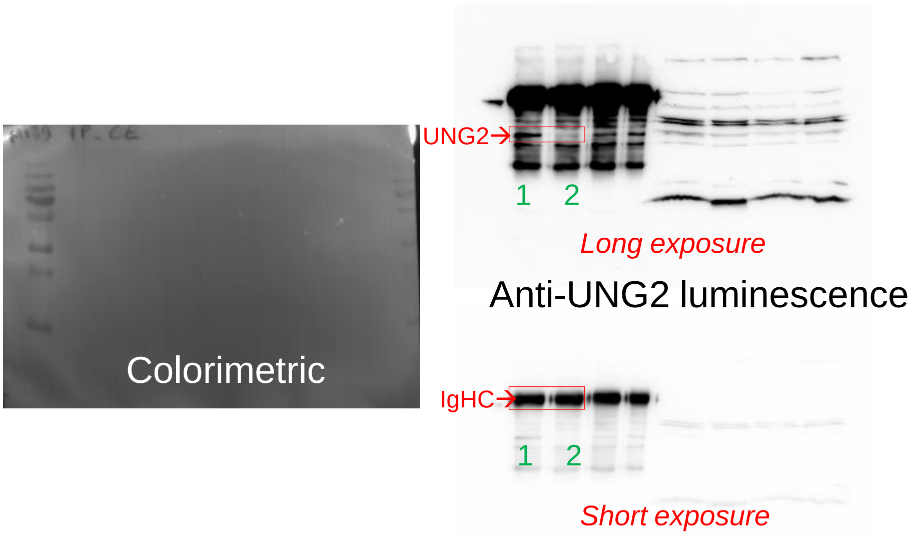

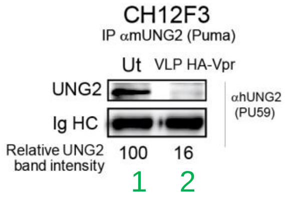

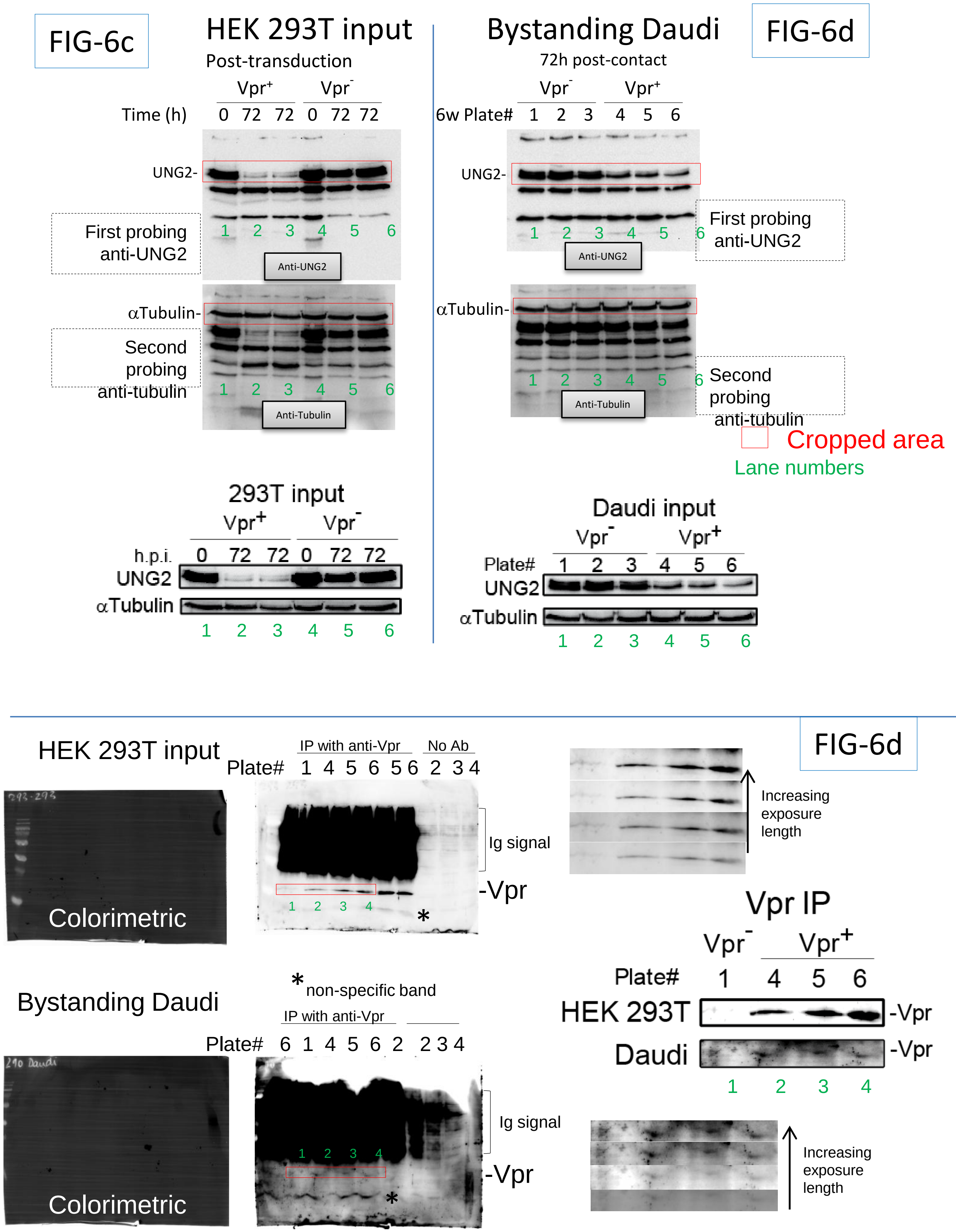
FIG-6c
HEK 293T input
Post-transduction
Vpr+ Vpr-
Time (h) 0 72 72 0 72 72
UNG2-
First probing anti-UNG2
1 2 3 4 5 6
Anti-UNG2
αTubulin-
Second probing anti-tubulin
1 2 3 4 5 6
Anti-Tubulin
Bystanding Daudi
72h post-contact
FIG-6d
Vpr- Vpr+
6w Plate# 1 2 3 4 5 6
UNG2-
First probing anti-UNG2
1 2 3 4 5 6
Anti-UNG2
αTubulin-
Second probing anti-tubulin
1 2 3 4 5 6
Anti-Tubulin
Cropped area
Lane numbers
293T input
Vpr+ Vpr-
h.p.i. 0 72 72 0 72 72
UNG2
αTubulin
1 2 3 4 5 6
Daudi input
Vpr- Vpr+
Plate# 1 2 3 4 5 6
UNG2
αTubulin
1 2 3 4 5 6
HEK 293T input
IP with anti-Vpr
No Ab
Plate# 1 4 5 6 5 6 2 3 4
293-293
Colorimetric
Ig signal
-Vpr
1 2 3 4
*
FIG-6d
Increasing exposure length
Vpr IP
Vpr- Vpr+
Plate# 1 4 5 6
HEK 293T -Vpr
Daudi -Vpr
1 2 3 4
Bystanding Daudi
* non-specific band
IP with anti-Vpr
Plate# 6 1 4 5 6 2 2 3 4
290 Daudi
Colorimetric
Ig signal
-Vpr
1 2 3 4
*
Increasing exposure length

FIG-6e

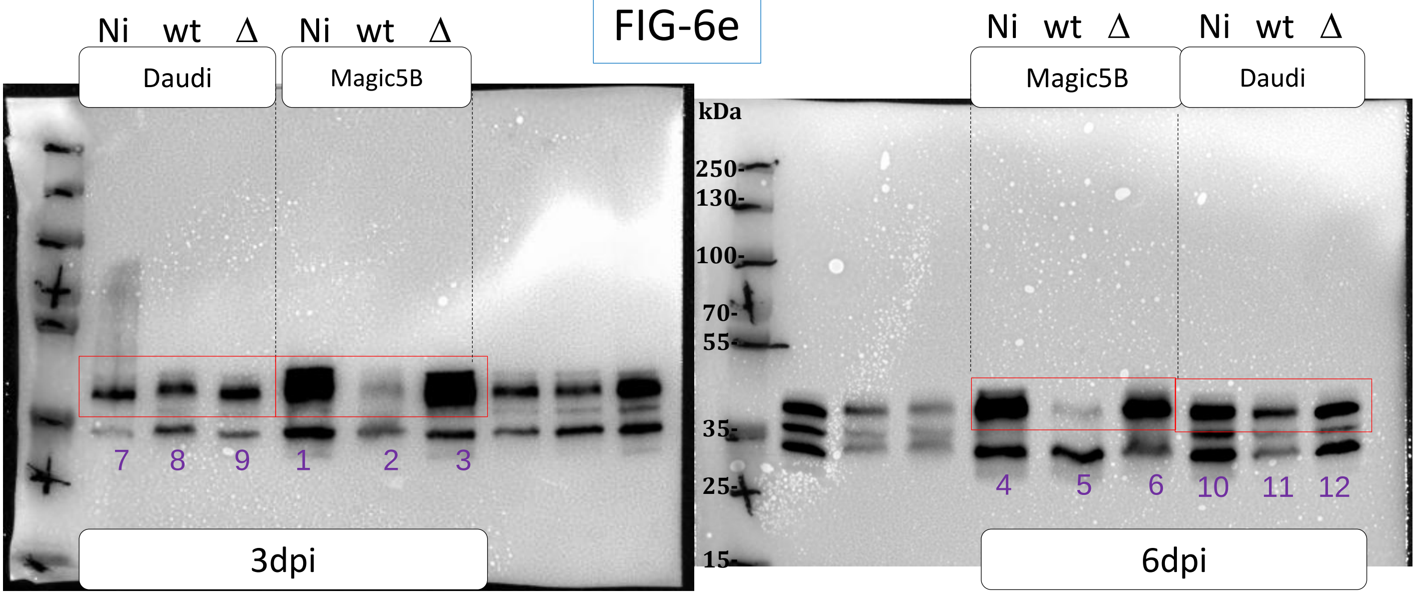

1st probing: Anti-UNG2 luminescence merged with colorimetric

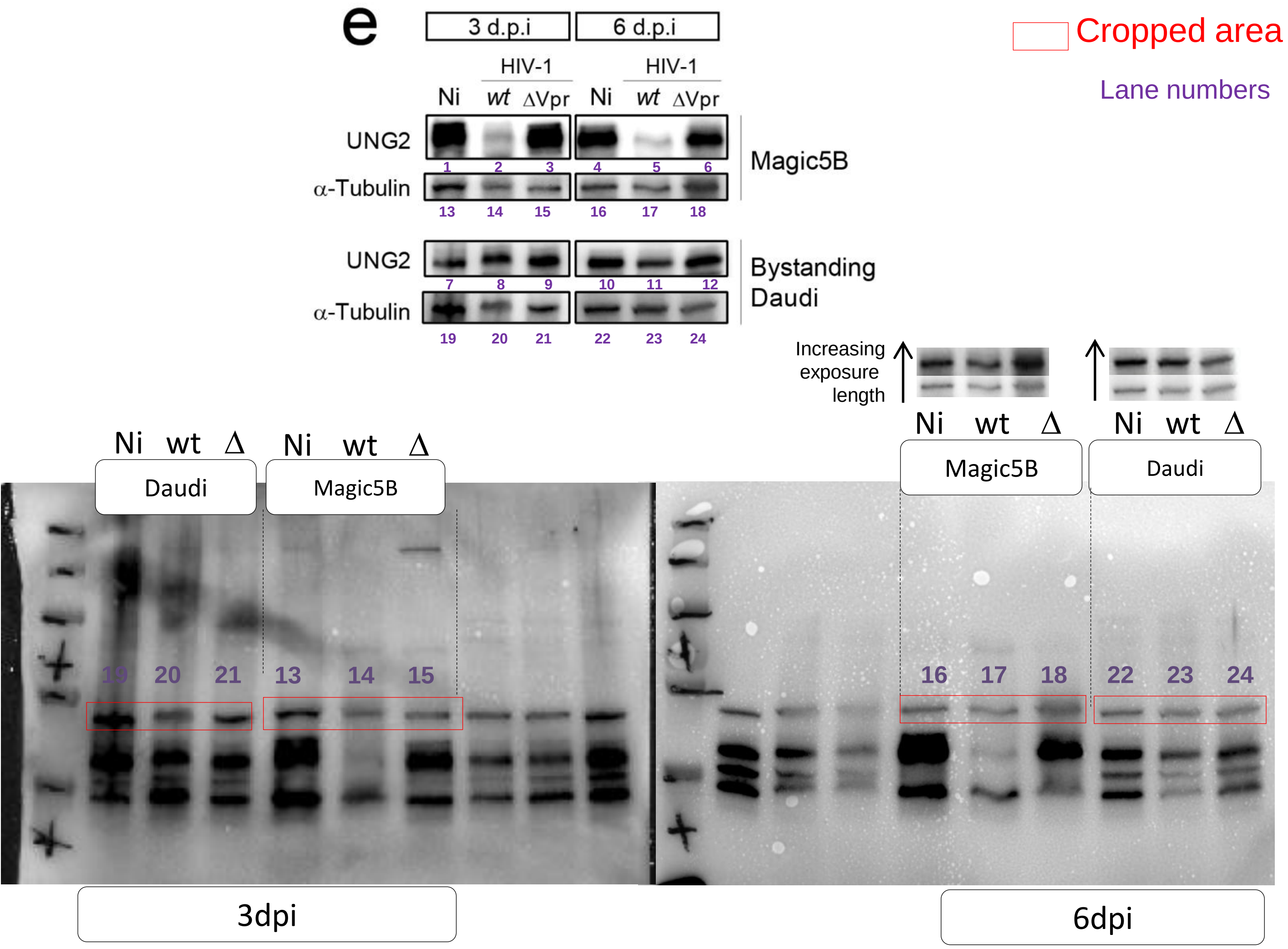

2nd probing: Anti-Tubulin luminescence merged with colorimetric
